# Clinical Outcomes and Predictors of Mortality in Variceal Upper Gastrointestinal Bleeding: A Retrospective Study From a Tertiary Care Hospital

**DOI:** 10.7759/cureus.104845

**Published:** 2026-03-08

**Authors:** Mudassir Raza, Mansoor Ul Hassan, Maira Zaheer, Dalvir Soor, Rida Ilyas, Hinza Farooq, Nizar Haddad

**Affiliations:** 1 Rheumatology, University Hospital Limerick, Limerick, IRL; 2 Internal Medicine, Shalamar Hospital, Lahore, PAK; 3 Acute Medicine, Basildon University Hospital, Mid and South Essex NHS Foundation Trust, Basildon, GBR; 4 Gastroenterology, James Cook University Hospital, Middlesbrough, GBR; 5 Internal Medicine, Services Hospital, Lahore, PAK; 6 Internal Medicine, Basildon University Hospital, Mid and South Essex NHS Foundation Trust, Basildon, GBR

**Keywords:** cirrhosis, endoscopy, hematemesis, upper gastrointestinal bleeding, variceal

## Abstract

Background

Variceal upper gastrointestinal bleeding (UGIB) is a life-threatening complication of portal hypertension and cirrhosis, associated with high morbidity and mortality. The objective of this study was to evaluate the clinical outcomes and identify independent predictors of six-week mortality in patients presenting with variceal UGIB at a tertiary care center, while providing locally representative data from a large cohort in a low- to middle-income country to strengthen region-specific evidence.

Methodology

This retrospective observational study was conducted at Shalamar Hospital, Lahore, Pakistan, from June 2020 to June 2025. It included 355 patients admitted with endoscopically confirmed variceal UGIB. Data regarding demographics, clinical presentation, laboratory parameters, endoscopic findings, treatment modalities, and outcomes were collected from hospital records.

Results

The mean age of patients was 52.3 ± 12.8 years, with 59.7% male patients. Most patients (83.9%) had known cirrhosis, and hematemesis was the most frequent presentation (69.0%). Endoscopic variceal ligation was the commonest intervention (76.3%). Initial hemostasis was achieved in 86.5% of patients. Rebleeding occurred in 20.8%, while the mean hospital stay was 6.9 ± 3.4 days. The overall six-week mortality was 21.1%. Independent predictors of mortality included rebleeding (AOR: 3.91; 95% CI: 2.10-7.30; p < 0.001), Child-Pugh class C (AOR: 2.84; 95% CI: 1.52-5.30; p = 0.001), and hemodynamic instability at admission (AOR: 2.76; 95% CI: 1.44-5.28; p = 0.002).

Conclusion

Variceal UGIB continues to pose a major clinical challenge, with significant mortality despite effective endoscopic and pharmacological interventions. Advanced liver dysfunction, rebleeding, and shock at presentation strongly predict poor outcomes.

## Introduction

Acute upper gastrointestinal bleeding (UGIB) is a medical emergency with a reported mortality ranging from 2% to 10% [1]. In the United States, the annual incidence of hospitalization for UGIB is approximately 160 per 100,000 persons, resulting in a substantial economic burden due to high direct hospital care costs [2]. The majority of acute UGI bleeding cases are non-variceal in origin, accounting for nearly 80%-90% of presentations [3].

Variceal UGIB, however, remains one of the most severe and life-threatening complications encountered in gastroenterology and hepatology. It occurs primarily as a consequence of portal hypertension, most commonly secondary to liver cirrhosis [4]. The condition is particularly feared due to its abrupt onset, massive blood loss, and high risk of early mortality despite advances in modern medical and endoscopic management. Variceal bleeding places a significant burden on healthcare systems worldwide, with a considerable proportion of patients with chronic liver disease requiring emergency hospitalization and urgent endoscopic interventions like endoscopic variceal ligation (EVL), endoscopic injection sclerotherapy (EIS), and balloon tamponade [5].

Variceal UGIB holds major clinical significance because of its close association with cirrhosis and the natural progression of portal hypertension. Esophageal varices develop in nearly 50% of patients with cirrhosis, and once present, the risk of rupture increases with rising portal pressure [6]. The likelihood of bleeding is influenced by variceal size, the presence of red wale signs, and the severity of underlying hepatic dysfunction. Rupture of varices often manifests as massive hematemesis or melena, frequently leading to hemodynamic instability and shock. Without prompt and effective intervention, mortality during the acute phase remains high [7].

Despite improvements in therapeutic strategies, mortality associated with acute variceal hemorrhage remains substantial. Current estimates suggest that approximately 15%-20% of patients die within six weeks of the initial bleeding episode [8]. Outcomes are determined not only by successful control of hemorrhage but also by the severity of underlying liver disease, commonly assessed using prognostic models such as the Child-Turcotte-Pugh (CTP) classification and the Model for End-Stage Liver Disease (MELD) score. Patients with decompensated cirrhosis, recurrent infections, renal dysfunction, or hepatocellular carcinoma have particularly poor prognoses, with higher risks of rebleeding and mortality [9].

Management strategies of variceal bleeding are guided by international consensus recommendations, including the Baveno VI and VII guidelines, which emphasize early resuscitation, vasoactive therapy, prophylactic antibiotics, and urgent EVL as first-line treatment [5,10]. High-risk patients, such as those with Child-Pugh class C cirrhosis or ongoing bleeding, may benefit from early or preemptive transjugular intrahepatic portosystemic shunt (TIPS) placement [10]. Unlike non-variceal UGIB, variceal bleeding is strongly influenced by portal hypertension and underlying liver disease, making disease-specific risk stratification and management critical.

The management of variceal bleeding has evolved significantly over recent decades. The introduction of vasoactive agents such as terlipressin, somatostatin, and octreotide, combined with prophylactic antibiotics and endoscopic therapy, has led to improved bleeding control and survival [11]. Endoscopic variceal ligation has largely replaced sclerotherapy due to its superior efficacy and lower complication rates. Nevertheless, rebleeding remains a major challenge, occurring in up to 30% of patients during the early period following the index bleed [12]. In refractory cases, TIPS serves as an effective salvage therapy, although its use may be limited by cost, availability, and the risk of hepatic encephalopathy, particularly in low- and middle-income settings [13].

Beyond short-term mortality, recurrent bleeding episodes, repeated hospitalizations, and progressive hepatic dysfunction significantly impair patients’ quality of life. The economic impact is also considerable, as affected patients often require intensive care support, multiple endoscopic procedures, and long-term secondary prophylaxis which include repeated EVL, non-selective beta-blocker therapy, or combination therapy [14]. In low- and middle-income countries, limited access to timely endoscopy, vasoactive medications, and blood products further worsens outcomes, contributing to disparities in survival when compared with well-resourced healthcare systems. Preventive strategies, including early risk stratification and appropriate secondary prophylaxis, therefore play a crucial role in improving clinical outcomes [15].

The aim of this study was to evaluate the clinical outcomes and identify independent predictors of six-week mortality in patients presenting with variceal upper gastrointestinal bleeding at a tertiary care center, while providing locally representative data from a large cohort in a low- to middle-income country, to strengthen region-specific evidence.

## Materials and methods

Study design and setting

This retrospective observational study was conducted at Shalamar Hospital, Lahore, Pakistan, over a five-year period from June 2020 to June 2025. A total of 355 patients presenting with variceal upper gastrointestinal bleeding were included. Data were obtained retrospectively from hospital medical records.

Inclusion and exclusion criteria

The study included adult patients aged 18 years or older who were admitted with acute upper gastrointestinal bleeding and had endoscopically confirmed esophageal or gastric varices. Eligible patients were required to have a documented history of cirrhosis or portal hypertension. Patients were excluded if they had non-variceal causes of UGIB, such as peptic ulcer disease, erosive gastritis, or Mallory-Weiss tear. Additionally, patients with incomplete medical records of treatment or clinical outcomes were excluded from the study. Six-week mortality data were obtained through a review of hospital electronic medical records, discharge summaries, hospital readmission records and, follow-up clinic documentation. Patients for whom this information could not be obtained were classified as “lost to follow-up” and were excluded from the analysis.

Data collection

Following approval from the institutional ethics committee, data were retrieved from hospital medical records, endoscopy registers, and patient files. Collected variables included demographic characteristics (age, gender), clinical presentation (hematemesis, melena, and hemodynamic status at admission), laboratory parameters (hemoglobin level, platelet count, liver function tests, and international normalized ratio), and endoscopic findings.

Information regarding management strategies, including the use of vasoactive agents, endoscopic variceal ligation, sclerotherapy, balloon tamponade, and transjugular intrahepatic portosystemic shunt, was documented. Clinical outcomes assessed included initial control of bleeding, occurrence of rebleeding, length of hospital stay, and mortality within six weeks of the index bleeding episode.

The primary outcome was all-cause mortality within six weeks following the index episode of variceal upper gastrointestinal bleeding. Secondary outcomes included rebleeding events, prolonged hospital stay (defined as hospitalization exceeding seven days), and the need for rescue therapy such as TIPS.

Rebleeding

Rebleeding was defined as recurrence of hematemesis and/or melena after initial hemostasis, a drop in hemoglobin ≥2 g/dL within 24 hours not explained by fluid resuscitation or phlebotomy, endoscopic confirmation of variceal bleeding, occurring within six weeks of the index bleeding episode.

Statistical analysis

Data were entered and analyzed using IBM SPSS Statistics, version 26 (IBM Corp., Armonk, NY). Continuous variables, including age, hemoglobin level, and duration of hospital stay, were summarized as mean ± standard deviation or median with interquartile range, depending on data distribution. Categorical variables were presented as frequencies and percentages.

The chi-square test was used to compare categorical variables. Variables with a p-value ≤0.20 on univariate analysis were entered into a multivariate binary logistic regression model to identify independent predictors of six-week mortality. Adjusted odds ratios (AORs) with 95% confidence intervals (CIs) were calculated. A p-value ≤0.05 was considered statistically significant.

For multivariate analysis, variables with a p-value ≤0.20 on univariate analysis were considered for inclusion. Continuous variables, including hemoglobin, platelets, creatinine, and MELD score, were initially evaluated; MELD was dichotomized at ≥18 for model stability and clinical interpretability. Multicollinearity between Child-Pugh class and MELD was assessed using the variance inflation factor (VIF), with all VIF values <2, indicating no significant multicollinearity. A backward stepwise likelihood ratio method was applied to construct the final model. Given 75 events (deaths), the final model was limited to three predictors (Child-Pugh class C, rebleeding, and hemodynamic instability) to maintain an events-per-variable (EPV) ratio >20, consistent with recommended logistic regression guidelines.

## Results

A total of 355 patients were included in the analysis. The mean age was 52.3 ± 12.8 years, and 212 patients (59.7%) were male. Most patients had a known diagnosis of cirrhosis (83.9%), while 16.1% were newly diagnosed at the time of admission.

Hematemesis was the most frequent presenting symptom (69.0%), followed by melena (51.3%); 20.3% of patients presented with both. Hemodynamic instability at admission was observed in 30.4% of cases. The mean hemoglobin level was 8.2 ± 2.4 g/dL, and the median platelet count was 86 ×10³/µL (IQR: 58-120). Regarding liver disease severity, 18.6% of patients were classified as Child-Pugh class A, 42.8% as class B, and 38.6% as class C [16,17]. The median MELD score was 17 (IQR: 13-21) (Table 1) [18].

**Table 1:** Baseline characteristics of patients (n = 355) MELD: Model for End-Stage Liver Disease

Variable	Value
Age (years), mean ± SD	52.3 ± 12.8
Male gender, n (%)	212 (59.7)
Female gender, n (%)	143 (40.3)
Known cirrhosis, n (%)	298 (83.9)
Newly diagnosed cirrhosis, n (%)	57 (16.1)
Hematemesis, n (%)	245 (69.0)
Melena, n (%)	182 (51.3)
Hematemesis + melena, n (%)	72 (20.3)
Hemodynamic instability at admission, n (%)	108 (30.4)
Hemoglobin (g/dL), mean ± SD	8.2 ± 2.4
Platelet count (×10³/µL), median (IQR)	86 (58–120)
Child–Pugh class A, n (%)	66 (18.6)
Child–Pugh class B, n (%)	152 (42.8)
Child–Pugh class C, n (%)	137 (38.6)
MELD score, median (IQR)	17 (13–21)

Endoscopic evaluation revealed esophageal varices in 85.1% of patients and gastric varices in 14.9%. EVL was the most commonly performed intervention (76.3%), while 13.8% underwent sclerotherapy. Balloon tamponade was required in 4.2%, and 5.1% of patients underwent TIPS as rescue therapy. Almost all patients received vasoactive therapy (98.0%), and 93.8% were administered prophylactic antibiotics (Table 2).

**Table 2:** Endoscopic and therapeutic interventions (n = 355) TIPS: transjugular intrahepatic portosystemic shunt

Variable	n (%)
Esophageal varices	302 (85.1)
Gastric varices	53 (14.9)
Endoscopic variceal ligation	271 (76.3)
Sclerotherapy	49 (13.8)
Balloon tamponade	15 (4.2)
TIPS performed	18 (5.1)
Vasoactive therapy	348 (98.0)
Prophylactic antibiotics	333 (93.8)

Initial hemostasis was achieved in 307 patients (86.5%). Rebleeding within six weeks occurred in 74 patients (20.8%). The mean duration of hospital stay was 6.9 ± 3.4 days, with 27.3% requiring prolonged hospitalization (more than seven days). The overall six-week mortality was 21.1% (Table 3).

**Table 3:** Clinical outcomes (n = 355)

Outcome	Value
Initial hemostasis achieved, n (%)	307 (86.5)
Rebleeding within six weeks, n (%)	74 (20.8)
Hospital stay (days), mean ± SD	6.9 ± 3.4
Prolonged stay (>7 days), n (%)	97 (27.3)
Six-week mortality, n (%)	75 (21.1)

On univariate analysis, Child-Pugh class C, MELD score ≥18, hemodynamic instability at admission, and rebleeding were significantly associated with six-week mortality.

Multivariate logistic regression identified rebleeding (AOR: 3.91; 95% CI: 2.10-7.30; p < 0.001), Child-Pugh class C (AOR: 2.84; 95% CI: 1.52-5.30; p = 0.001), and hemodynamic instability at admission (AOR: 2.76; 95% CI: 1.44-5.28; p = 0.002) as independent predictors of mortality (Table 4).

**Table 4:** Predictors of six-week mortality (n = 355) *Statistically significant at p ≤ 0.05; logistic regression model adjusted for age, gender, and cirrhosis status. MELD: Model for End-Stage Liver Disease; AOR: adjusted odds ratio

Variable	OR (95% CI)	p-value	AOR (95% CI)	p-value
Child–Pugh class C	3.25 (1.89–5.60)	<0.001*	2.84 (1.52–5.30)	0.001*
MELD score ≥18	2.72 (1.61–4.58)	<0.001*	1.59 (0.87–2.89)	0.12
Hemodynamic instability	3.43 (2.02–5.81)	<0.001*	2.76 (1.44–5.28)	0.002*
Rebleeding	4.62 (2.68–7.94)	<0.001*	3.91 (2.10–7.30)	<0.001*

Rebleeding was significantly more frequent among patients aged ≥60 years, those with Child-Pugh class C, MELD score ≥18, and hemodynamic instability at presentation. Variceal location was not significantly associated with rebleeding risk (Table 5).

**Table 5:** Factors associated with rebleeding *Statistically significant at p ≤ 0.05; chi-square test applied. MELD: Model for End-Stage Liver Disease

Variable	Rebleeding (n = 74), n (%)	No Rebleeding (n = 281), n (%)	χ² value	p-value
Age ≥60 years	26 (35.1)	61 (21.7)	5.32	0.021*
Male gender	47 (63.5)	165 (58.7)	0.50	0.48
Child–Pugh class C	41 (55.4)	96 (34.2)	9.60	0.002*
MELD score ≥18	43 (58.1)	91 (32.4)	13.85	<0.001*
Hemodynamic instability	32 (43.2)	76 (27.0)	6.46	0.011*

Prolonged hospitalization was significantly associated with older age, hemodynamic instability, rebleeding, advanced liver disease, higher MELD scores, and the need for TIPS. Gender showed no significant association (Table 6).

**Table 6:** Factors associated with prolonged hospital stay (more than seven days) *Statistically significant at p ≤ 0.05; chi-square test applied. MELD: Model for End-Stage Liver Disease; TIPS: transjugular intrahepatic portosystemic shunt

Variable	Prolonged stay (n = 97), n (%)	Short stay (n = 258), n (%)	χ² value	p-value
Age ≥60 years	32 (33.0)	55 (21.3)	4.84	0.028*
Male gender	60 (61.9)	152 (58.9)	0.23	0.63
Hemodynamic instability	42 (43.3)	66 (25.6)	10.60	0.001*
Rebleeding	39 (40.2)	35 (13.6)	27.80	<0.001*
Child–Pugh class C	46 (47.4)	91 (35.3)	4.02	0.045*
MELD score ≥18	51 (52.6)	83 (32.2)	13.50	<0.001*
TIPS required	12 (12.4)	6 (2.3)	14.20	<0.001*

## Discussion

In this retrospective study of 355 patients presenting with variceal upper gastrointestinal bleeding, we observed that despite the availability of standard therapies such as vasoactive agents, endoscopic interventions, and prophylactic antibiotics, mortality and morbidity remained substantial. The six-week mortality rate of 21.1% observed in our cohort is consistent with international reports indicating mortality rates of 15%-25% following acute variceal hemorrhage, depending on patient characteristics and access to advanced treatment. These findings reinforce that variceal bleeding remains a major cause of mortality among patients with cirrhosis and portal hypertension.

Advanced liver disease emerged as a critical determinant of poor outcomes. In our cohort, a large proportion of deaths occurred among patients with Child-Pugh class C cirrhosis, highlighting the importance of hepatic reserve in determining survival following an acute bleeding episode. This finding emphasizes the need for early referral to transplant centers and consideration of advanced therapeutic strategies such as transjugular intrahepatic portosystemic shunt in carefully selected patients [19]. Similar associations between advanced cirrhosis and increased mortality have been reported in previous studies.

Rebleeding was another key factor associated with adverse outcomes in this study. Most rebleeding episodes occurred in patients with severe cirrhosis and hemodynamic instability at presentation, supporting the concept that host-related factors, in addition to therapeutic limitations, contribute significantly to recurrence of bleeding [20]. Although endoscopic variceal ligation was the most frequently employed intervention (76.3%) and achieved satisfactory initial hemostasis in the majority of patients, those requiring salvage therapies such as balloon tamponade or TIPS represented a subgroup with markedly higher morbidity and mortality [21].

Despite the proven efficacy of TIPS in controlling bleeding and improving survival in high-risk patients, its use remains limited in many low- and middle-income settings because of high cost, limited availability, and concerns regarding procedure-related complications. These barriers were also evident in our setting and likely contributed to poorer outcomes among severely ill patients. Our findings support existing evidence advocating for early-TIPS strategies in appropriately selected high-risk patients, which may reduce rebleeding and improve survival when resources allow [22].

Given the high six-week mortality observed in our cohort, effective prevention and management strategies are critical to improving outcomes in variceal UGIB [23]. Primary prophylaxis with non-selective beta-blockers and endoscopic variceal ligation in patients with high-risk varices can reduce the risk of first bleeding. In the acute setting, rapid hemodynamic resuscitation, early administration of vasoactive agents, prophylactic antibiotics, and urgent endoscopic intervention are essential to achieve initial hemostasis [5]. Secondary prophylaxis, combining endoscopic variceal ligation with beta-blockers, helps prevent rebleeding, while salvage therapies such as TIPS are indicated in patients with recurrent bleeding or failure of standard interventions [10]. Early risk stratification using clinical and laboratory parameters can identify high-risk patients who may benefit from preemptive escalation of care. Nevertheless, mortality remains substantial among patients with advanced liver disease, hemodynamic instability at presentation, or early rebleeding, highlighting the ongoing need for timely intervention, close monitoring, and improved access to advanced therapies in resource-limited settings.

When compared with data from high-income countries, the mortality rate observed in our cohort was slightly higher. This difference may be explained by disparities in healthcare infrastructure, delayed patient presentation, limited access to specialized services such as TIPS, and the high prevalence of advanced cirrhosis at admission in our population [24]. Advanced liver disease itself remains a major constraint to survival, even when optimal medical and endoscopic therapies are available.

The strengths of this study include a relatively large sample size and a comprehensive evaluation of clinical, laboratory, and therapeutic predictors of outcomes, providing a realistic reflection of routine clinical practice. However, this retrospective, single-center study was limited by the availability of medical records, incomplete laboratory and follow-up data, and inability to ascertain cause-specific mortality, so only all-cause six-week mortality is reported. This is a limitation, as the relative contribution of acute variceal bleeding versus complications of advanced liver disease (hepatic failure, sepsis, or encephalopathy) could not be assessed. Severely ill patients who died before undergoing endoscopy were not included, potentially underestimating mortality. Treatment protocols, including timing of endoscopy, use of vasoactive agents, and secondary prophylaxis, varied, reflecting real-world practice but possibly influencing outcomes. Finally, residual confounding from unmeasured variables cannot be excluded despite multivariate adjustment.

## Conclusions

Variceal upper gastrointestinal bleeding remains a life-threatening complication of portal hypertension, associated with substantial morbidity and mortality despite advances in medical and endoscopic therapy. In this study, the six-week mortality rate was 21.1%, highlighting the significant clinical burden of this condition. Rebleeding, advanced liver disease (Child-Pugh class C), and hemodynamic instability at presentation were strongly associated with increased mortality. Although endoscopic variceal ligation combined with vasoactive therapy achieved effective initial hemostasis in most patients, recurrent bleeding continued to adversely affect survival. Early risk stratification, prompt resuscitation, antibiotic prophylaxis, and timely endoscopic intervention remain essential components of management. Secondary prophylaxis with beta-blockers and endoscopic ligation, along with consideration of advanced therapies such as TIPS in high-risk patients, may further improve outcomes. Future efforts should focus on earlier detection of cirrhosis, improved access to specialized care, and optimized preventive strategies to reduce mortality.
